# Case Study: Chronic Recurrent Multifocal Osteomyelitis in the Femoral Diaphysis of a Young Female

**DOI:** 10.1155/2012/515761

**Published:** 2012-02-28

**Authors:** Jeffrey S. Quon, Anne K. Dzus, David A. Leswick

**Affiliations:** ^1^Department of Medical Imaging, Ottawa Hospital, University of Ottawa, Civic Campus, Ottawa, ON, Canada K1N 6N5; ^2^Division of Orthopedic Surgery, Department of Surgery, Royal University Hospital, University of Saskatchewan, Saskatoon, SK, Canada S7N 0W8; ^3^Department of Medical Imaging, Royal University Hospital, University of Saskatchewan, Saskatoon, SK, Canada S7N 0W8

## Abstract

Chronic recurrent multifocal osteomyelitis (CRMO) is relatively uncommon. Even though the name suggests it is the result of infection, this is not likely the case. Instead it is more likely the result of genetic, autoimmune, or autoinflammatory causes. Although CRMO has a benign course and responds well to anti-inflammatory medications, it can have a very aggressive clinical and imaging presentation overlapping with infectious osteomyelitis and malignancy. Therefore, radiologists and clinicians need to be aware of its clinical and imaging presentation to avoid morbidity associated with more aggressive treatment. We present the case of a ten-year-old female with CRMO as a solitary expansile-mixed lytic and sclerotic lesion in the distal femoral diaphysis. The diaphyseal location and mixed lytic and sclerotic appearance are less common and have an aggressive imaging appearance. We also review the pathophysiology, imaging findings, and therapeutic approach to this uncommon but clinically important condition.

## 1. Case Presentation

A 10-year-old girl presented to the orthopedic service with a two-year history of “aching-type” pain over the anterior distal right thigh. The pain did not radiate, was worse with rest, and was rated as approximately 5/10 in intensity. The discomfort waxed and waned but recently had increased in intensity and frequency. Ibuprofen helped relieve some of the pain. The patient was otherwise healthy with no history of fevers, chills, or weight loss. She had no history of previous surgeries nor a family history of bone or joint abnormalities, including tumors.

Examination revealed a tender area of fusiform swelling in the distal right thigh centered just above the superior border of the patella with no erythema or knee effusion. Her right lower extremity was neurovascularly intact with the exception of decreased knee reflex compared to the left side. There was no inguinal lymphadenopathy. Her gait was normal. The patient's white blood count (WBC) was normal, erythrocyte sedimentation rate (ESR) was slightly elevated at 27 mm/hr (0–20 mm/hr), and C-reactive protein (CRP) was normal at 6.1 mg/L (0–7.0 mg/L).

**Figure 1:**
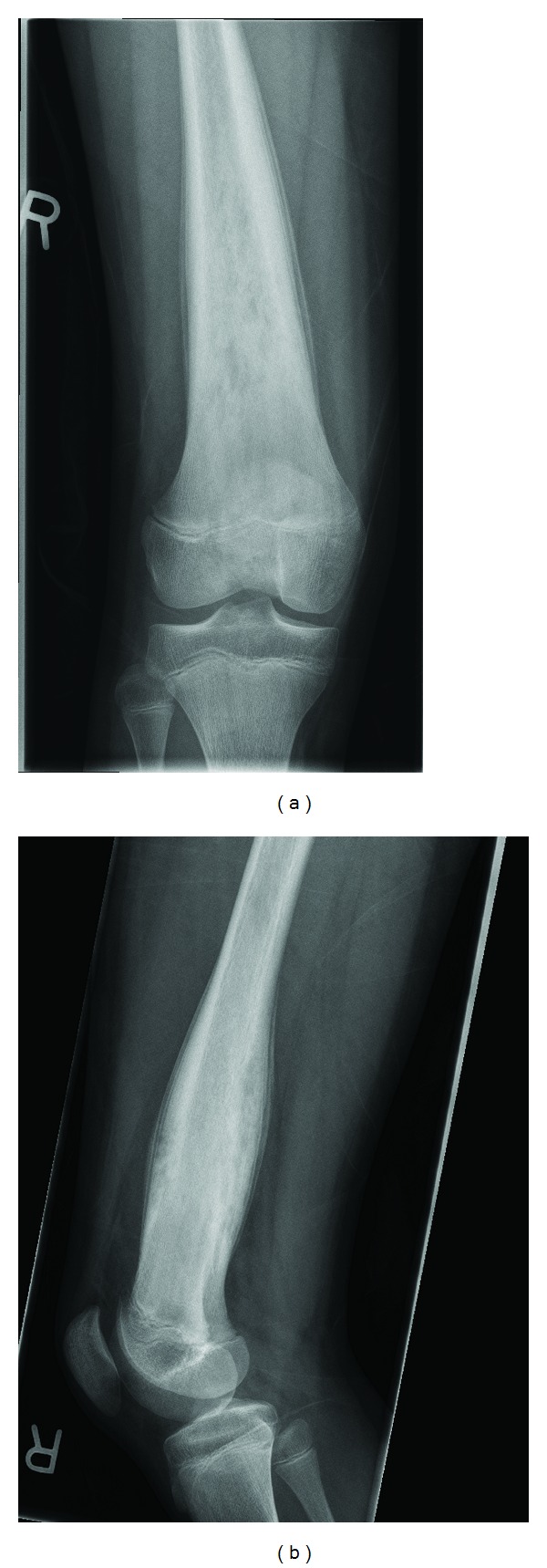
Frontal (a) and lateral (b) radiographs of the distal right femur. The lesion is shown as an expansile, fusiform mass in the distal femur, away from the growth plate. Medullary involvement is shown as patchy, moth-eaten lucencies. Smooth laminated onion-skin like periosteal reaction is also seen.

Radiographs of the femur and knee (Figure 1) revealed an expansile, moth-eaten appearing bony lesion with poorly defined margins centered in the distal diaphysis of the femur. There was significant “onion skin” periosteal reaction. The lesion spared the metaphysis and did not involve the growth plate or the epiphysis. Computed tomography (CT) of the chest and femur, technetium bone scan of the whole body, and MRI of the femur and thigh were all performed within 48 hours of presentation.

**Figure 2:**
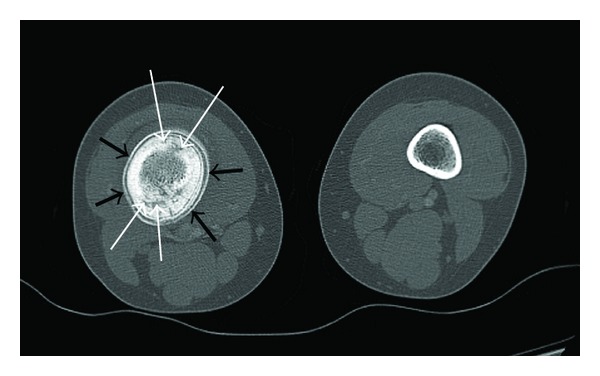
Axial 1.25 mm image enhanced CT through the lesion. The right femoral lesion is well shown on CT, including cortical thickening, cortical lucencies (thin white arrow), and periosteal reaction (black arrows). Note the size discrepancy between the right and left femurs in keeping with the clinically seen asymmetry and fusiform swelling.

CT of the distal femur following IV contrast (Figure 2) revealed similar findings to the radiographs. Of note is the fusiform enlargement, cortical lucencies, and periosteal reaction centered in the distal diaphysis. CT of the chest revealed no abnormalities.

**Figure 3:**
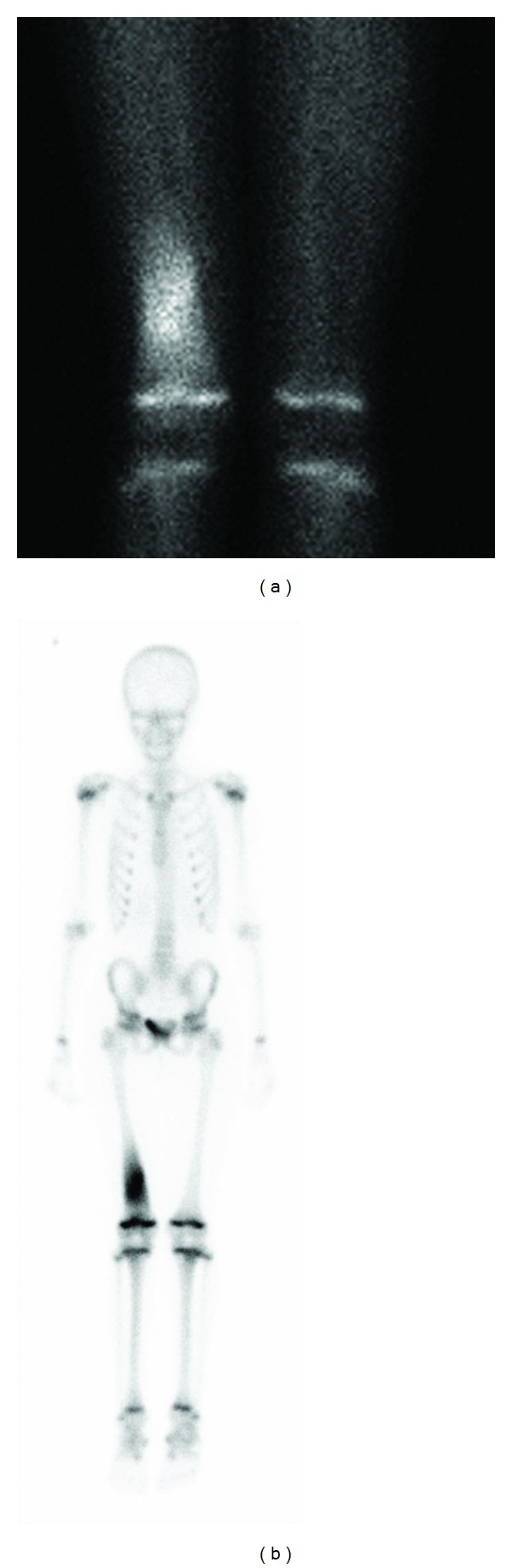
Frontal projection femoral blood pool (a) and 2-hour delayed whole-body (b) images from 99 mTc MDP bone scan. Intense activity is present at the right distal femoral lesion. There were no other active sites, confirming this to be a solitary lesion.

A technetium bone scan (Figure 3) showed the lesion to have intense activity on both blood pool and delayed static views. The activity was isolated to the diaphysis with no extension to the growth plate or epiphysis. This was confirmed to be a solitary lesion.

**Figure 4:**
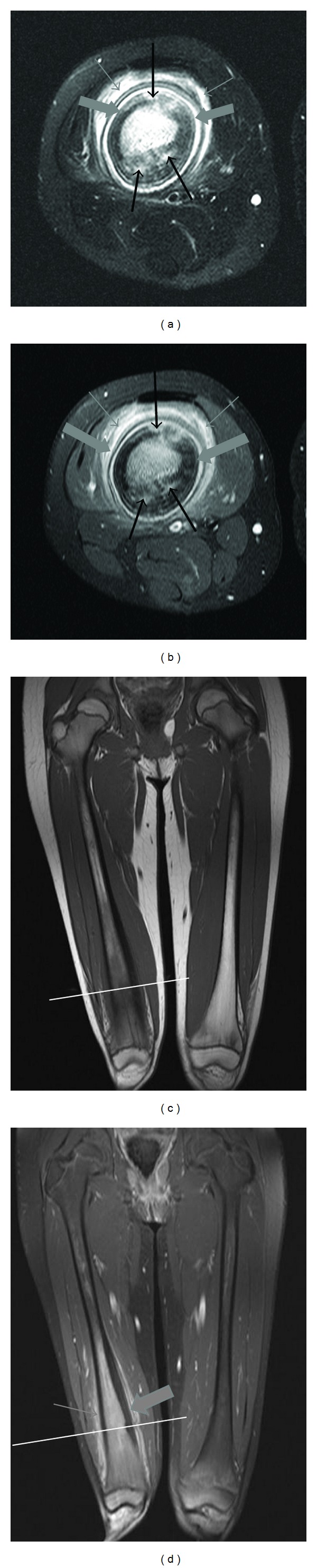
Axial (a-b) MRI through the right thigh with coronal (c-d) MRI images through both thighs. Displayed axial images are from the level of the white lines in (c) and (d). Sequences are as follows: (a) axial T2 fat saturated (FS) (TE 93 ms. TR 5670 ms.), (b) axial T1FS with gadolinium (TE 17 ms. TE 730 ms.), (c) coronal T1 (TE 14 ms. TR 543 ms.), and (d) coronal T1FS with gadolinium (TE 14 ms. TR 460 ms.). The axial T2 FS image displays the edematous high signal in the medullary space, patchy high signal cortical lesions (black arrows), cortical thickening and periosteal reaction (thick grey arrows), and subtle surrounding soft tissue edema (thin grey arrows). Coronal T1 (c) shows the low signal within the medullary space, a characteristic feature of involvement. After gadolinium (b,d) there is intense enhancement of the marrow, periosteum (thick grey arrows), with some enhancement also seen in the bordering musculature (thin grey arrows).

MRI (Figure 4) again showed the mass to be expansile, centered within the distal diaphysis with periosteal reaction. Intrinsically the mass was T1 dark relative to yellow marrow and T2 bright especially with fat saturation. There was enhancement throughout the ill-defined mass and some edema and enhancement was seen in the adjacent musculature.

The case was reviewed with a pediatric orthopedic tumor surgeon who suggested that this may be CRMO based on the two-year history and the CT findings. The patient started on a trial of Naproxen prior to biopsy. Two weeks following presentation, an open bone biopsy was performed via an approach anterolateral to the iliotibial band. Both intramedullary and cortical bone samples from the lesion were obtained and sent for pathological analysis. Histological analysis revealed reparative and reactive sclerotic bone with fibrosis and scattered chronic inflammatory cells consistent with chronic osteomyelitis.

The patient had immediate significant relief of her symptoms with Naproxen (an NSAID). She continued on regular Naproxen for about 6 months and was symptom-free, which allowed her to resume full sporting activities. At 18 months after biopsy, attempts at discontinuing her Naproxen resulted in recurrent pain at the site.

## 2. Discussion

Chronic Recurrent Multifocal Osteomyelitis (CRMO) was first described in 1972 in four patients with “subacute and chronic “symmetrical” osteomyelitis” by Giedion et al. [1, 2]. However, the first report may actually date back to a sclerosing form of osteomyelitis described by Garre in 1893 [3]. Many of the early reports came from Scandinavian countries, but CRMO has since been reported in most parts of the world [4]. There have been over 200 cases of CRMO described in the literature, but the incidence remains unknown [2, 5]. It occurs mostly in children and adolescents of European descent [6] but has been diagnosed in other ethnicities and in adults [5, 7–9]. Females are affected more often than males [4, 5, 7].

Despite being recognized as a clinical entity for more than thirty years, the origin and pathophysiology of CRMO remains unknown [4–6, 10]. Hematogenous spread of infection seems unlikely as pathogens are rarely cultured, with occasional positive cultures favoured to be the result of contamination rather than true infection [5]. Several observations including concordant monozygotic twins and affected siblings suggest that genetic factors may play a role. CRMO is associated with several autoimmune diseases including inflammatory bowel disease, Wegner's granulomatosis, and psoriasis [5]. It has also been reported as associated with or as a pediatric variant of SAPHO syndrome (synovitis, acne, pustulosis, hyperostosis, and osteitis) [2, 5]. Girschick et al. [10] has suggested that further research into the areas of autoinflammatory, autoimmunity, errors of metabolism, and postinfectious reactive inflammation may yield some answers to CRMO's pathogenesis [10].

Clinical presentation is variable depending on the specific site of involvement. Although the disease title includes the term multifocal, CRMO lesions may be solitary or multiple and synchronous or metachronous [2, 5]. The clinical course is often long-lasting with episodes of exacerbation [4]. Regional symptoms include localized pain, tenderness and swelling over the involved bones [5, 9]. Systemic effects can include occasional low-grade fever and slight malaise [5]. Laboratory findings are nonspecific, with many patients having elevated ESR and CRP, but normal WBC count [2, 5]. CRMO is generally thought of as a self-limited disease with the majority of lesions resolving without complication [5, 7]. Although limited, the duration of symptoms can be very prolonged in the range of 7 to 25 years [5].

As in the presented case, the long bones are a common site of involvement. Other sites including the clavicles, spine, pelvis, and sacroiliac joints; the anterior chest; scapula; metatarsals and metacarpals; phalanges; tarsal bones; mandible have also been reportedly involved to a lesser degree [2, 6, 11]. For the purpose of this report, we shall focus on the imaging of long bone lesions.

 Radiographic evaluation of CRMO lesions can be characteristic but not pathognomonic [5]. Early stage CRMO may show decalcification or osteolysis, while later stages of the disease may present as hyperostosis and sclerosis [6]. At any stage, periosteal reaction may also be visualized. Tubular bone lesions are most often found at the metaphyses of long bones but can extend to the diaphyses and occasionally the epiphyses [2, 5]. Initial radiographs usually demonstrate metaphyseal disease [4], which frequently manifests as eccentric lytic lesions adjacent to the growth plate with a sclerotic rim separating it from the underlying bone and limited or no periosteal reaction [5]. This metaphyseal disease later fills in and heals with sclerosis and later normalization of the radiographic appearance. As in our presented case, the lesions can also involve the diaphysis, which may result from spread of an earlier metaphyseal lesion [5]. Diaphyseal lesions are characterized by lytic destructive areas and periosteal reaction, which heal with sclerosis and hyperostosis [5, 9]. Recurrent active lesions will progressively lay down further bone adding to the expansion and sclerotic appearance [10] (Figure 1). Active diaphyseal lesions may show small lytic areas with regions of new bone formation [5], a finding that is better seen on the CT (Figure 2) of our presented case.

Focal pathology in the skeletal system can be best detected using bone scintigraphy [1] although the result may be negative if the inflammatory activity is low [5, 6]. Bone scintigraphy can identify all symptomatic lesions and frequently clinically silent lesions as well. This may help in the diagnosis of CRMO [2].

Computed tomography has a limited role in the diagnosis of CRMO [6]. CT findings parallel those described under radiographic assessment, with the advantage of detecting subtle bone destruction, especially in anatomically difficult sites like the sternum, spine, and pelvis. Sclerosis and periosteal reaction may also be seen [5]. As shown in the presented case, small lytic areas of active disease can be identified on the CT (Figure 2). Its major drawback is the significant radiation exposure, which must be considered especially in children [6]. A CT of the chest was also obtained in our patient to rule out metastatic disease as Ewing's sarcoma was part of the working differential diagnosis.

MRI may be useful to further characterize lesions including bone marrow and adjacent soft tissue involvement as well as for surveillance [5, 6, 9]. Appearance on MRI will depend on whether lesions are in an active or reparative phase [5]. Although normal bone marrow signal is variable in children depending on erythropoietic activity, diaphyseal marrow is typically fatty in adolescents appearing as bright on T1 and intermediate signal on T2-weighted images [12]. During active inflammation, MR imaging shows findings typical of marrow edema, which appears hypointense on T1-weighted images and hyperintense on T2-weighted images (Figure 4) [5, 6, 9, 13]. During quiescent disease, signal intensity will decrease on both T1- and T2-weighted sequences because of sclerosis. MRI will also show cortical thickening and periosteal reaction [6]. One of the key aspects of MRI imaging is the absence of abscess or sinus tract formation as this helps to discriminate against bacterial osteomyelitis [5]. However, CRMO may have surrounding tissue inflammation [5], which can be seen as adjacent soft tissue increased T2 signal and enhancement, as in the presented case (Figure 4). MRI helps determine the best location for biopsy [6] and has the added benefit of not exposing pediatric patients to ionizing radiation [5, 6]. In indeterminate cases, whole-body MR imaging may be useful for the detection of CRMO because it is more likely to show abnormalities compared to lab tests or other radiological investigations [13].

Positron emission tomography (PET) has been used clinically to detect chronic osteomyelitis, but its use in CRMO has not been described [2]. Similarly, ultrasound has been used in imaging bacterial osteomyelitis, but its use in the investigation of CRMO has not been documented [2].

CRMO often remains a diagnosis of exclusion between infectious osteomyelitis and neoplasm as there is often overlap of clinical and imaging findings [4, 10, 14]. Neoplasms such as osteosarcoma, Ewing's sarcoma, neuroblastoma, rhabdomyosarcoma, leukemia, Langerhans' cell histiocytosis, osteoid osteoma, and osteoblastoma are often part of the differential diagnosis [6, 10]. In our presented case, the differential diagnosis included bacterial osteomyelitis and Ewing's sarcoma. Bacterial osteomyelitis was determined less likely based on the atypical location and the absence of abscess or sinus tract formation, while biopsy ruled out Ewing's sarcoma.

The primary means of diagnosing CRMO relies on clinical presentation, plain radiography, and bone scintigraphy [2]. When the diagnosis is uncertain, CT and MRI are useful to further define the disease extent [13]. If CRMO is the most likely consideration, CT and MRI should be used only for radiographically occult lesions identified on bone scan or lesions that appear atypical [2]. In a 1998 publication, Handrick et al. suggested the following imaging strategy if the diagnosis of CRMO is considered based on clinical presentation: (1) radiographs of the symptomatic lesions with or without ultrasound, (2) bone scintigraphy to identify additional lesions (i.e., multifocal disease), (3) radiographs of any additional lesions shown on bone scan, (4) MR imaging for further assessment of lesions that are detected on bone scan but appear normal on radiograph and may be clinically suspect.

 The treatment of CRMO may involve various therapeutic agents and/or operative procedures [2]. Antibiotics are often used for empiric therapy if a bacterial etiology is suspected. However, once the diagnosis of CRMO is made, antibiotics should be discontinued as they are ineffective [2, 7]. Nonsteroidal anti-inflammatory drugs (NSAIDs) have shown variable benefit in CRMO therapy and are considered the best choice for treatment [2, 5, 10]. Although corticosteroids have been shown to have some effect on the disease course, their side effects render them a less than ideal choice [5]. Other alternatives may include interferon-*α* [2, 5], interferon-*γ* [2, 5], bisphosphonates [5], sulfasalazine [5, 10], methotrexate [5], colchicines [2], and gammaglobulin [10]. Widespread use of surgical intervention has not been reported although partial or complete claviculectomy of clavicular lesions has been documented with some success [2, 5].

CRMO is thought of as an uncommon disease although the exact incidence remains unknown. Even though it is a benign and self-limited condition, which often responds to NSAID treatment, its radiological appearance can be aggressive, overlapping with bacterial osteomyelitis and neoplasm. Awareness of this condition and correlation with provided clinical history can help the radiologist and clinicians offer this diagnosis, potentially sparing the patient from unnecessary invasive testing and aggressive management [5, 14].
